# Relationship Between Infrared Thermography and Functional Parameters in the Lower Limbs of Hemiplegic Patients

**DOI:** 10.3390/life15040542

**Published:** 2025-03-26

**Authors:** Alessio Cabizosu, Alberto López-López, Daniele Grotto, Josefina Maria Vegara-Meseguer

**Affiliations:** 1 THERMHESC Group, Ribera Hospital de Molina San Antonio, Catholic University of Murcia (UCAM), 30107 Murcia, Spain; dgrotto3@alu.ucam.edu; 2 Astrapace Institute, Association for the Treatment of Persons with Cerebral Palsy and Related Pathologies, 30107 Murcia, Spain; alberto.lopez@astrapacemurcia.es; 3 Computer Engineering Department, Catholic University San Antonio of Murcia (UCAM), 30107 Murcia, Spain; jmvegara@ucam.edu

**Keywords:** thermography, brain damage, modified Ashworth scale, timed up and go, pressure pain threshold

## Abstract

Introduction: Reliable objective and non-invasive assessments of myotendinous alterations in patients with muscle tone disorders secondary to brain damage represent an important challenge in health science. The aim of this study was to observe the relationship between the skin temperature and the functional response in the triceps suralis of hemiplegic patients in relation to the healthy control group. Methods: A descriptive observational study was conducted based on the STARD recommendations. A total of 26 volunteers, 13 participants with unilateral motor impairment and 13 healthy patients, participated and completed the study. Intragroup and intergroup clinical thermography tests were performed, and the results were compared in relation to the timed up and go test, pain threshold to pressure, and modified Ashworth scale. Results: Statistically relevant differences (*p* < 0.01) could be observed between the two groups in each test performed. Thermographic analysis revealed a difference in temperature between the healthy and affected sides in the inter- and intra-group comparisons. It was possible to observe statistically significant differences (*p* < 0.01) between limbs in the brain damage group (the side affected was at a lower temperature), while no such differences were observed between limbs in the healthy control group (*p* > 0.05). Conclusions: Our results confirmed that clinical thermography could be a potentially useful tool in the assessment of both structural and functional alterations of the musculoskeletal system in patients with chronic brain damage.

## 1. Introduction

In patients with muscle tone disorders secondary to brain damage, myotendinous alterations of the triceps suralis are a consequence of the tension or spasticity generated on the soft tissues via indirect orders from the central nervous system (CNS) [1]. Spasticity, a hallmark of upper motor neuron syndrome, is characterized by a velocity-dependent increase in muscle tone due to hyperexcitability of the stretch reflex [2]. This excessive tone can lead to myotendinous adaptations, including stiffness, shortening, and fibrotic changes, which in turn contribute to biomechanical restrictions in the lower limbs. In severe cases, these structural modifications can significantly impair gait and postural control, thereby diminishing the overall quality of life [3,4,5,6].

The understanding of neuromuscular responses in such patients has been the focus of extensive research aiming to elucidate the physiological mechanisms underlying these alterations to in order to provide useful information about the assessment, possible treatment, and follow-up of patients affected by this chronic condition [7,8]. From a neuromechanical perspective, spasticity-induced changes involve alterations in muscle architecture, extracellular matrix remodeling, and modifications in viscoelastic properties, which can further influence motor performance and rehabilitation outcomes [9].

However, due to the great difficulty in standardizing and quantifying accurately, objectively, and reliably the functional response of patients in relation to the degree of soft tissue involvement, it is not possible to quantify the functional response of patients in a standardized manner [10]. It has not been conclusively determined which diagnostic test may be the best option for clinical and functional assessment of neuromuscular sequelae.

From a medical diagnostic point of view, in the neurological field, neuroimaging has indeed promoted the study and macroscopic analysis of the different brain regions; however, from a functional diagnostic point of view, there are still no known objective, fast, non-invasive, and low-cost tools that can quantify the functional and metabolic impairment associated with these structural changes. In fact, functionally, neurological patient assessment is mainly based on quantitative numerical scales [11], although according to some authors there are controversies about their reliability [12].

Thermoregulation, a crucial physiological process involving the autonomic nervous system, plays a fundamental role in maintaining homeostasis in response to metabolic activity and tissue perfusion. In neurological conditions, alterations in thermoregulation may reflect underlying functional impairments in peripheral circulation and muscle metabolism [13].

In order to suggest a solution to the existing conflict between tissue involvement and functional response, recent studies have suggested that clinical thermography could be a useful and reliable tool for the assessment of the physiological, metabolic, and functional state of the myotendinous system in patients with neuromuscular disorders, obtaining good correlations between thermographic patterns and complementary tests [14]. Infrared thermography is an imaging technique that allows the determination of the surface temperature of an object; it has greater accuracy when the object has a high emissivity level i.e., the capacity to emit its own infrared radiation [15,16].

The progressive use of this tool in the neurological field is due to the fact that infrared thermography is an objective, easy-to-use, low-cost, and non-invasive imaging technique that allows the analysis of infrared radiation emitted by the human body and, after converting it into a thermal pattern, provides a visual image on a color scale that correlates with skin temperature [17,18]. Because less molecular agitation at the tissue level releases less infrared radiation, which translates into a lower skin thermal pattern, the use of infrared thermography has been the subject of several studies assessing muscle atrophy and weakness in the neurological area [19,20,21]. There exists a certain consensus that in patients with brain damage the temperature of the affected side is lower than that of the healthy side, due to the tissue and vascular changes inherent to the pathology that affect tissue molecular aggression [14]. However, the latest studies published on this subject show limitations in terms of the homogeneity of the sample, which, according to the authors, has an impact on the results obtained. Thus, despite the promising results obtained, specific research is needed to confirm the hypothesis that clinical thermography could be used as a diagnostic tool for musculoskeletal disorders associated with motor disability.

To this end, the aim of this study was to observe the relationship between the skin temperature and the functional response in the triceps suralis of hemiplegic patients in relation to the healthy control group. The novelty of this work lies in the variety of diagnostic tests performed, in the homogeneity of the sample, and in the compliance with the most current protocols on clinical thermography. The hypothesis of this work is that, in hemiplegic patients, differences can be observed in the emission of infrared radiation between the healthy side and the affected side. We also believe that the thermal response of the affected side will be lower with respect to both sides compared to the control group. The thermographic images could be correlated with the functional tests in relation to the muscular sequelae of brain damage.

## 2. Material and Methods

### 2.1. Study Design

A descriptive observational study was carried out based on Standards for the Reporting of Diagnostic Accuracy Studies (STARD) recommendations [22]. Both healthy volunteers and patients with unilateral neuromuscular alterations secondary to brain damage participated in the study. All participants received an information sheet about the study procedures and signed an informed consent form. Two investigators specializing in brain injury classified the patients based on age, sex, BMI, and functional diagnosis (Table 1). The study was conducted in accordance with the Declaration of Helsinki for Research Involving Human Subjects [23]. The study was approved in October of 2021 by the ethics committee of the Catholic University San Antonio of Murcia (CE102107).

### 2.2. Participants

A total of 26 volunteers participated in this observational study, 13 participants with unilateral motor impairment and 13 healthy patients. No limits were set regarding sex, race, or BMI. The inclusion criteria were (1) healthy non-athletic participants over the age of majority for the healthy control group, (2) volunteers who had suffered brain damage with more than 5 years of evolution with functional impairment in only one hemi-body for the affected group. The following were excluded from the study: (1) Participants who were in a febrile state regardless of the group. (2) Those who had suffered a lower extremity injury 3 months prior to the study regardless of the group. (3) Those who were taking medication related to thermoregulatory changes. (4) People with intolerance to any of the tests or who presented any issue that negatively influenced the DITI results according to the investigators. (5) Volunteers who had been infected with SARS-CoV-2 within the last year. Of the 26 total participants, 13 were healthy, 8 patients had right spastic hemiplegia, and 5 had left spastic hemiplegia.

### 2.3. Familiarization

In this session, the study procedures were explained to the participants and information about compliance with the Tisem protocol was provided [24]. The volunteer’s suitability to participate in the study was confirmed and data about age, sex, BMI (using Tanita BC-545 Innerscan Segmental Body Composition [25,26]), and functional diagnosis were collected. In addition, participants were placed in the thermographic and PPT assessment position to familiarize them with the stimuli and distances. Finally, the phases and execution of the TUG test were explained.

### 2.4. Index Test: Thermal Data Acquisition

**Figure 1:**
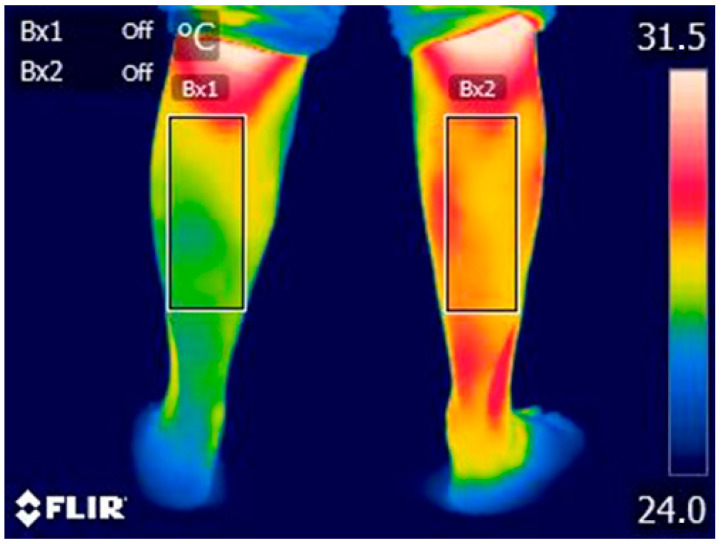
Clinical thermography image of a hemiplegic patient. BX1: Left leg, BX2: Right leg.

Infrared thermography (IRT) is a non-invasive imaging technique that detects infrared radiation emitted from the skin’s surface, providing a visual representation of temperature distribution. In medical research, particularly studies focusing on hemiplegic patients, IRT has been utilized to asses skin temperature variations and their relationship to functional parameters [27]. Infrared thermography results can be influenced by several factors that can affect skin temperature. To avoid mistaken apparent temperatures, some procedures have been taken into account in concordance with the literature recommendations [28,29]. Prior to the thermographic test, participants underwent acclimatization for 15 min in a 20 m^2^ room with a controlled ambient temperature of 22.0 ± 0.5 °C and a relative humidity 40 ± 0.5% [24]. Electronic devices and light sources were controlled to avoid interference in the acquisition of thermographic images. Infrared data were acquired using a FLIR E75 thermal camera (FLIR Systems, Inc., Wilsonville, OR, USA) with a pixel infrared resolution of 320 × 240, thermal sensitivity <0.04 °C, and with fixed thermal records from 20 °C to 120 °C. The emissivity was 0.98, as suggested by other authors [28,30,31]. The thermographic device was switched on 30 min before the first recording on a tripod with a 10–15 °C tilt at 1 m from the measurement site. Participants wore a T-shirt and shorts and were placed in a standing position in front of the thermographic device. For all thermographic measurements of the regions of interest (ROI), references proposed by other authors were used, Figure 1 [32]. The thermographic response was considered valid as an index test when there was a statistically significant difference between the two extremities in the affected group and between the affected side of the brain-damaged patients and either side of the control group. Researchers recorded and analyzed the images in a blinded manner to ensure reliability. Researchers performing the index test were blinded to the reference standards. Image processing was carried out using Flir IR Research software 4.1.

### 2.5. Reference Standard

All participants were assessed for sensory response via PPT [33] and motor response via MAS [11] and the TUG test [34], since all these tests have been validated and tested in these types of patients by other authors [14,35,36].

The PPT test was used as a reference standard to determine the difference in pain sensitivity between posterior lower extremities. The difference in sensitivity system between sides was considered when there was a greater capacity to withstand pressure before pain appeared. The algometer used for the PPT was the Wagner force TENTM FDX model (Greenwich, CT, USA). This model is intended for hand-held force testing, with a pistol grip handle operating on a rechargeable battery for portable use, and the margin of error is ±0.3%. The PTT protocol stated that patient had to inform the examiner as soon as discomfort appeared after pressure was applied at the level of the muscle belly of the inner calf. To avoid vascular interference that could alter the thermographic results, PPT was performed after the thermographic measurement and was expressed in Kg.

MAS scales were used as a reference standard to determine the difference in motor function because the purpose of this scale is to grade muscle spasticity. The scale is as follows. 0: No increase in muscle tone. 1: Slight increase in muscle tone, with a catch and release or minimal resistance at the end of the range of motion when an affected part(s) is moved in flexion or extension. 1+: Slight increase in muscle tone, manifested as a catch, followed by minimal resistance through the remainder (less than half) of the range of motion. 2: A marked increase in muscle tone throughout most of the range of motion but affected part(s) are still easily moved. 3: Considerable increase in muscle tone, passive movement difficult. 4: Affected part(s) rigid in flexion or extension [37].

The TUG test was performed according to protocols described by other authors [38] and was used as a reference standard to determine the normal or typical development of a motor task. A motor task was considered different when the time taken for it was found to be significantly different between groups. The timed up and go test is a clinical functional mobility assessment test that measures the time it takes a patient to stand up, walk 3 m, turn around, walk back, and sit down again. To start the TUG test, the patient will sit in a chair with arms resting on their legs and their back well supported; the test starts when the investigator clearly verbalizes “START” and starts the stopwatch. The stopwatch will stop when the patient sits down again after standing up, walking 3 m, turning around a cone, and returning to the starting position [39].

### 2.6. Statistical Analysis

Data analysis was performed using IBM SPSS Statistics, version 21. A descriptive analysis of the variables was conducted, presenting measures as mean ± standard deviation for quantitative variables. To assess whether there are significant differences between thermographic, sensitive, and motor responses between the two groups, Student’s *t*-test was performed for independent samples for those variables that were normally distributed according to the Shapiro–Wilk test (*p* > 0.05), while the Mann–Whitney U test was performed for those in which the null hypothesis of normality was rejected (*p* < 0.05). To corroborate the diagnostic power of the tests, we also checked whether there were statistically significant thermographic, sensory, and motor differences according to the laterality in each group, graphically representing which group these differences were associated with. For this, Student’s *t*-test was performed for paired samples in those variables normally distributed according to the Shapiro–Wilk test (*p* > 0.05) and the Wilcoxon test for those in which the null hypothesis of normality was rejected (*p* < 0.05). Cohen’s d effect sizes (ES) (95% confidence interval) were calculated for intragroup comparisons of temperature, algometry, modified Ashworth, and timed up and go test. Threshold values for the ES statistics were as suggested by other authors [25]. The term “affected side” in relation to the healthy control group corresponds to the results of the same side and the same region of interest as the pathological group, but in the control group. Therefore, despite being named as the affected side, it must be considered that it is a non-affected side. The level of statistical significance was set at *p* < 0.05.

## 3. Results

Table 2 shows the results obtained in the different tests, according to each group and each calf. From a descriptive point of view, it is possible to observe differences between the two groups in each test performed, with a lower temperature on the affected side of the pathological group compared to all other limbs, higher PPT and MAS values on the affected side of the pathological group compared to all other limbs, and higher TUG values in the pathological group compared to the control group.

### 3.1. Difference in Temperature, PPT, and MAS According to Group

#### 3.1.1. Affected Group

**Figure 2:**
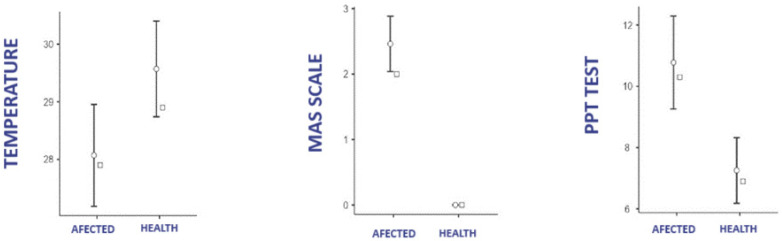
Difference in temperature, PPT, and MAS between extremities in the affected group.

Figure 2 shows the data for temperature, MAS scale, and PPT test, according to laterality in the affected group. The Shapiro–Wilk tests reported a violation of the normality assumption in the PPT test between the affected side and the healthy side of the pathological group, so differences in these variables were tested using the Wilcoxon test. Both Student’s *t*-test and the Wilcoxon test showed significant differences between the healthy side and the affected side in pathological patients in all three diagnostic tests (*p* < 0.001) ICC 95% −2.02, ICC 95% 0.97, ICC 95% 3.17

#### 3.1.2. Control Group

**Figure 3:**
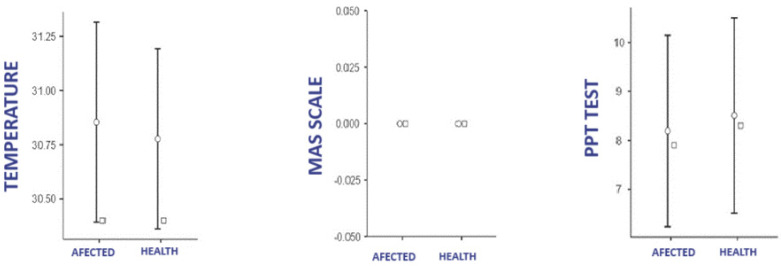
Difference in temperature, PPT, and MASS between limbs in the control group.

Figure 3 shows the data for temperature, MAS scale, and PPT test according to laterality in the health group. Shapiro–Wilk tests reported the normality of the data for temperature differences and PPT. In the MASS, no correlations could be shown, as none of the patients were pathological, so the result was equal to 0 in both extremities. Student’s *t*-test showed no significant difference in temperature and PTT between sides in the healthy group (*p* = 0.24) ICC 0.33, (*p* = 0.24) ICC −0.33.

### 3.2. Difference of Temperature, PPT y MAS Between Group

**Figure 4:**
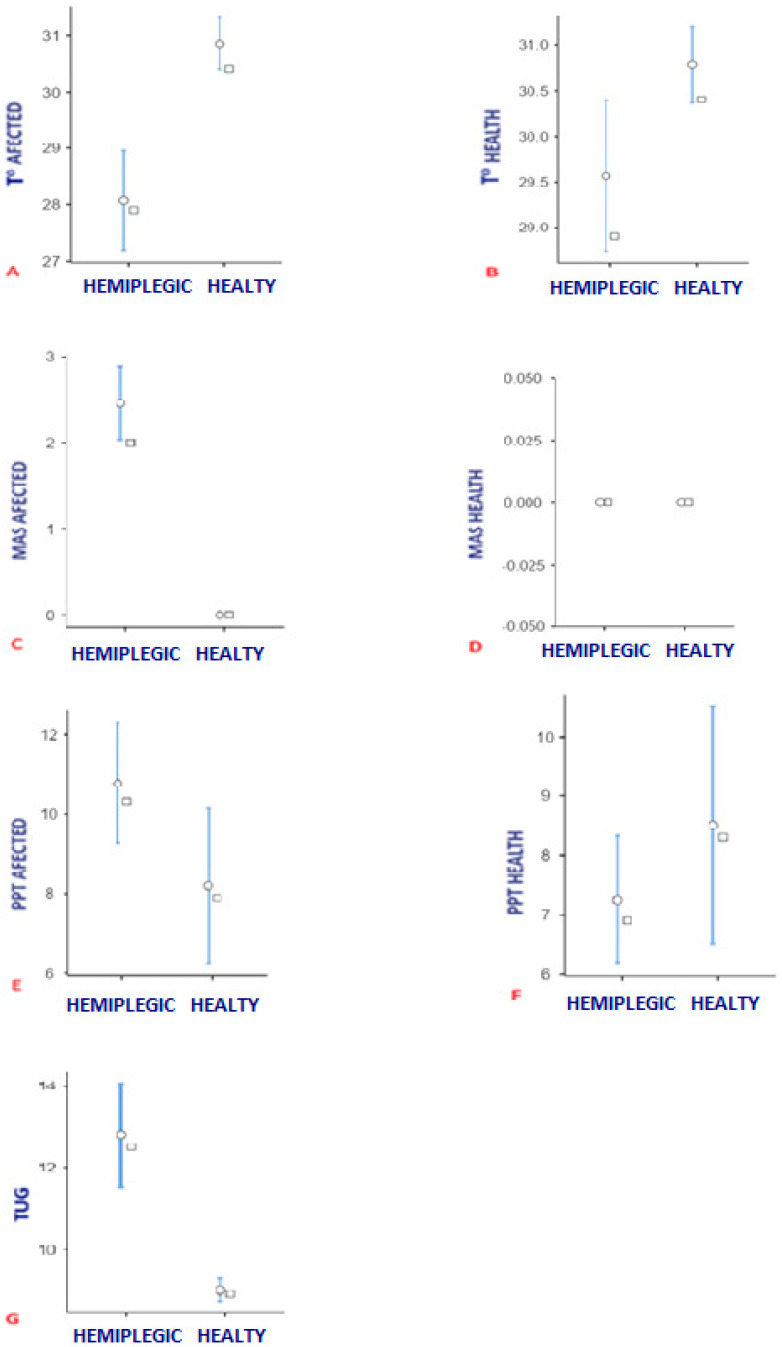
Difference in temperature, PPT, MAS, and TUG between groups. (**A**) Temperature difference between pathologic side in hemiplegic group and the same side in control group. (**B**) Temperature difference between healthy side in hemiplegic group and the same side in control group. (**C**) MAS scale difference between pathologic side in hemiplegic group and the same side in control group. (**D**) MAS scale difference between healthy side in hemiplegic group and the same side in control group. (**E**) PTT test difference between pathologic side in hemiplegic group and the same side in control group. (**F**) PTT test difference between healthy side in hemiplegic group and the same side in control group. (**G**) TUG test between pathologic group and control group.

Figure 4 shows the data for temperature, MAS scale, PPT test, and TUG test according to group. The Shapiro–Wilk tests reported a violation of the normality assumption in MAS between the affected side of the pathological group and the same side in the group of healthy patients (*p* < 0.05), and there was also no normality in the TUG test (*p* < 0.05); therefore, in these variables, the differences were tested using the Mann–Whitney U test, which showed statistically significant differences between these variables. In addition, Student’s *t*-test showed a significant difference in temperature between the affected side and the healthy side between the two groups, with (*p* < 0.01) ICC −2.1, and even between the healthy sides of both groups (*p* < 0.02) ICC −1.0.

## 4. Discussion

The aim of this study was to observe the relationship between the skin temperature and the functional response in the triceps suralis of hemiplegic patients in relation to the healthy control group. The results found in this study regarding the comparison between groups showed a statistically significant difference (*p* < 0.01) between temperature, PPT, and MAS in the posterior region of the affected side of the hemiplegic group patients compared to the same region in the control group patients and even between the healthy sides of both groups (*p* < 0.02) ICC −1.0. Furthermore, statistically significant differences (*p* < 0.01) were found between the control group and the pathological group in the TUG. Regarding the difference between limbs in each group, a statistically significant difference was observed between the pathological side and the non-pathological side in the brain-damaged group in all three tests (*p* < 0.01) ICC 95% −2.02, ICC 95% 0.97, ICC 95% 3.17, while no such difference between limbs could be observed in the control group (*p* = 0.24) ICC 0.33, (*p* = 0.24) ICC −0.33.

From a thermographic point of view, our results support previous studies in which it has been observed that the affected side in patients with brain damage has a lower skin temperature than the contralateral side [40,41]. In fact, in this study, 46.1% of the brain-injured group showed a ΔT between legs varying between 0.5 °C and 1 °C, 23% between 1.1 °C and 1.5 °C, and 38.4% had a ΔT of more than 1.5 °C. In the control group, however, 100% of the sample showed a side-to-side difference of less than 0.5 °C. These findings may be due to the fact that in patients with spasticity secondary to brain damage, there is an increased infiltration of intramuscular adipose tissue on the affected side compared to the healthy side, which can generate structural and vascular changes in the musculoskeletal system [42]. These changes may lead to a decrease in metabolic and vascular activity at the deep level, which is reflected in a decrease in the emission of infrared radiation at the cutaneous level by convection, something that does not happen in healthy patients; since as tissue metabolic and functional activity is respected in healthy patients, temperature differences of more than 0.5 °C should not be observed [43]. Another remarkable aspect of this work was that the authors found a statistically significant difference between the healthy side of patients with brain damage and the same side in the control group. Although, a priori, this may seem a contradiction in diagnostic terms, it should be noted that thermoregulatory processes are not generated only in a single limb but at a global level, so that data obtained on the contralateral side are possibly the result of systemic physiological processes and not an isolated response. The idea that thermoregulation is a complex and global process has already been observed by other authors in healthy patients, as it has been shown that, thermographically, the physiological and metabolic activation of a single side produces changes in the contralateral side at rest [44,45]. This could be generated at rest in patients with brain damage, as there is an underlying metabolic and physiological change due to the pathological state, which requires a systemic thermoregulatory mechanism. Since the thermal difference between sides is a characteristic and consolidated aspect in patients with brain damage, future studies could find out if a decrease in the temperature difference between the healthy and affected sides correlates with an improvement in motor and sensory functionalities. This could be of great interest for physiotherapists, who could use thermography as an element of evaluation and monitoring of therapeutic protocols.

In relation to the motor response measured with the MAS scale and TUG test between groups, our results support previous studies in which a direct relationship between these variables was observed in affected patients compared to healthy patients. The MAS scale measures joint stiffness to movement and has been used and validated in numerous studies, as greater stiffness corresponds to greater functional impairment [37], relating a higher MAS in the ankle flexor muscles of patients with chronic brain damage with a decrease in speed in the TUG test [46]. This could be due to the fact that when there appears to be limitations in the articular range of dorsiflexion and plantiflexion, specificity of movement and direction of rotation is lost, so that the time–distance relationship would be affected, although it should be noted that there is still no common consensus between studies of kinematic and kinetic variables in relation to spatio-temporal variables in this type of patient [47,48]. Future research could further investigate the relationship between the functional limitations of dorsiflexion and the length or cadence of the step in relation to the probable distance to determine whether these variables can be related to thermographic parameters. Descriptively, in our study, in the group where the motor tests showed worse results, a lower skin temperature is observed, due to, according to other authors, the muscular atrophy of the triceps suralis [49,50]. Notwithstanding, there is still no consensus due to the small number of related studies on the subject and the lack of comparability of the results between them due to the great differences in the measurement protocols and the type of sample [51]. This highlights the need for health professionals to use assessment and treatment techniques that aim to observe deep metabolic responses, and it is therefore extremely important to continue research in this area using clinical thermography, as it is a fast, reliable, inexpensive, and non-invasive technique that provides instant feedback on the responses obtained after the treatment or assessment carried out.

In relation to the results obtained in PPT test between the healthy and affected sides, our results support the findings obtained by other authors who showed higher results for pressure algometry in the affected side compared to the healthy side [52,53]. It is known from other authors that the spinothalamic pathway and the dorsal spinal pathway are the main pathways for pain, pressure, and temperature in the central nervous system [53,54]. Once at the cutaneous level, both superficial and deep, the information has been picked up by the main mechanoreceptors [54,55] and the signal ascends via different processes and pathways to the thalamus; from there, it is projected to the cerebral cortex in the different Brodmann’s areas responsible for metabolizing and processing the cutaneous tactile, thermal, and proprioceptive stimuli [56]. To this end, numerous authors relate sensory and motor alterations to each other in this type of patient, justifying that sensory alterations secondary to brain damage could be the cause of alterations in normal motor control [57,58]. However, there is no clear and conclusive information on whether it is the sensory system that generates a deficit in distal motor activity or whether distal impairments prevent perfect central encoding of sensory patterns, thus limiting mobility. Nevertheless, there is evidence that pathologies related to central sensitization are associated with increased pressure sensitivity [59], whereas patients affected by peripheral nerve diseases affecting the sensory part may have a higher pain threshold due to sensory deficit [60]. It should be taken into account that in both circumstances a process of functional limitation of motor activity may be generated due to the fact that movement stimulates pain and, consequently, the tendency of patients is to move less, or that the lack of sensitivity produces limited information at a central level, which results in a reduced motor response, so further studies are needed to clarify the relationship between pain and mobility in relation to cutaneous thermal changes.

Future studies could deepen this knowledge, clarifying the role of thermography in relation to sensory and motor alterations. It would be interesting for this purpose to consider variables such as the brain area affected in relation to the time of involvement in order to provide more exhaustive information on the relationship between cortical involvement vs. peripheral response. According to the results obtained in this preliminary study, the thermal challenge between sides recorded with infrared thermography on the back of the leg could be an indicator of the level of functional involvement in patients with hemiplegia secondary to brain damage. However, it should be considered that, although the small sample size of this study may represent, a priori, a limiting factor, recruiting patients belonging to the same geographical area and having a diagnosis of brain damage with preserved motor and cognitive abilities has been the main difficulty encountered in this study. In addition, although the small sample size may have influenced the study’s statistical power, we believe that the results of this study are reliable and can be of support for the development of future research. Nevertheless, future studies with larger sample sizes and more specific diagnostic and functional tools are needed to help understand how different skin thermal changes may relate to different degrees of neuromuscular involvement.

The recording of thermal levels could be of interest for predicting the motor function of the affected structures, thus reaching a consensus on the anatomical region affected and the degree of affectation. To this end, future studies could associate these thermographic results with the metabolic effects generated at the muscular level in muscle fatigue and complement the functional assessment with electromyography, perhaps in a larger sample of patients. This could not only facilitate diagnostic work but also open new lines of pharmacological and therapeutic assessment. If the results obtained in this study were proven in future research, the use of clinical thermography as an objective diagnostic assessment tool in different areas of health science could be strongly justified.

## 5. Conclusions

Our results confirmed that clinical thermography could be a potentially useful tool in the assessment of both structural and functional alterations of the musculoskeletal system in patients with chronic brain injury. However, future studies with larger sample sizes are needed to corroborate and support the results obtained in this preliminary study.

## Figures and Tables

**Table 1:** Baseline Characteristics.

Characteristics	Brain Damage Group (N = 13)	Control Group (N = 13)
Sex Woman Man	46% 53%	46% 53%
Age	30.5 ± 6.8	24.6 ± 3.8
BMI	26 ± 6.5	26.4 ± 5.7
Affectionate side Right Left	61% 39%	/ /

Values are expressed as percentages (%) or as mean ± standard deviation/healthy.

**Table 2:** Test results.

	Group	Mean ± SD	IQR [25–75%]
T° Affected Calf	Hemiplegic	28.0 ± 1.6	26.9–29.5
	Healthy	30.8 ± 0.8	30.3–31.6
T° Health Calf	Hemiplegic	29.5 ± 1.5	28.5–30.8
	Healthy	30.7 ± 0.7	30.2–31.3
PPT Affected	Hemiplegic	10.7 ± 2.7	8.7–11.8
	Healthy	8.1 ± 3.5	5.3–9.30
PPT Health	Hemiplegic	7.2 ± 1.9	5.9–8.20
	Healthy	8.5 ± 3.6	6.2–9.70
MAS Affected	Hemiplegic	2.4 ± 0.7	2.0–3.0
	Healthy	0.0 ± 0.0	0.0–0.0
MAS Health	Hemiplegic	0.0 ± 0.0	0.0–0.0
	Healthy	0.0 ± 0.0	0.0–0.0
TUG	Hemiplegic	12.7 ± 2.3	11.6–13.8
	Healthy	8.9 ± 0.5	8.6–9.40

Values are expressed as mean ± standard deviation.

## Data Availability

Data generated or analyzed during this study are included in this article.

## Abbreviations

ATM	Atmosphere
BMI	Body Mass Index
CNS	Nervous central system
DITI	Digital infrared thermal imaging
ΔT	Difference of temperature
MAS	Modified Ashworth scale
PPT	Pressure pain threshold
ROI	Region of interest
STARD	Standards for the Reporting of Diagnostic Accuracy Studies
TISEM	Thermographic Imaging in Sports and Exercise Medicine
TUG	Timed up and go
T°	Temperature
